# Effect of D2R, NMDAR and CB1R genetic variants associated with cannabis use and childhood trauma in first-episode psychosis in a Brazilian population

**DOI:** 10.1192/j.eurpsy.2023.584

**Published:** 2023-07-19

**Authors:** C. M. Loureiro, F. Corsi-Zuelli, H. A. Fachim, R. Shuhama, P. R. Menezes, C. F. Dalton, P. Louzada-Junior, S. I. N. Belangero, F. B. Coeli-Lacchini, G. P. Reynolds, R. Lacchini, C. M. Del-Ben

**Affiliations:** ^1^Neuroscience and Behavior, Ribeirão Preto Medical School, University of São Paulo, Ribeirão Preto; ^2^Population Mental Health Research Centre, São Paulo, Brazil; ^3^University of Manchester, Manchester, United Kingdom; ^4^Preventive Medicine, University of São Paulo, São Paulo, Brazil; ^5^Biomolecular Sciences Research Centre, Sheffield Hallam University, Sheffield, United Kingdom; ^6^Internal Medicine, Ribeirão Preto Medical School, University of São Paulo, Ribeirão Preto; ^7^Morphology and Genetics, Universidade Federal de São Paulo, São Paulo; ^8^Ribeirao Preto College of Nursing, University of São Paulo, Ribeirão Preto, Brazil

## Abstract

**Introduction:**

Gene-environment interactions increase psychosis risk (Gayer-Anderson *et al*. Soc Psychiatry Psychiatr Epidemiol 2020; 55(5):645-657). However, identifying the genetic variants involved and how they interact with environmental risk factors underlying psychosis remains challenging.

**Objectives:**

To investigate whether there are gene-environment interactions in the relationships of childhood trauma, lifetime cannabis use, and single nucleotide variants (SNVs) of dopamine D2 receptor (D2R: *DRD2*), N-methyl-d-aspartate receptor (NMDAR: *GRIN1*, *GRIN2A* and *GRIN2B*) and cannabinoid receptor type 1 (CB1R: *CNR1*) with psychosis.

**Methods:**

In a population-based case-control study nested in an incident study (STREAM, Brazil) (Del-Ben *et al*. Br J of Psychiatry 2019; 215(6):726-729), part of the EU-GEI consortium (Gayer-Anderson *et al*. Soc Psychiatry Psychiatr Epidemiol 2020; 55(5):645-657), 143 first-episode psychosis patients and 286 community-based controls of both sexes aged between 16 and 64 years were included over a period of 3 years. Twenty-three SNVs of D2R (rs1799978, rs7131056, rs6275), NMDAR (*GRIN1*: rs4880213, rs11146020; *GRIN2A*: rs1420040, rs11866328; *GRIN2B*: rs890, rs2098469, rs7298664), and CB1R genes (*CNR1*: rs806380, rs806379, rs1049353, rs6454674, rs1535255, rs2023239, rs12720071, rs6928499, rs806374, rs7766029, rs806378, rs10485170, rs9450898), were genotyped from peripheral blood DNA using a custom Illumina HumanCoreExome-24 BeadChip. Environmental adversities were evaluated using the Cannabis Experience Questionnaire (Di Forti et al. The Lancet Psychiatry 2009; 6(5):427–436) and the Childhood Trauma Questionnaire (Grassi-Oliveira et al. Rev Saude Publica 2006; 40(2):249-55). Associations between SNVs and environmental risk factors were performed using the nonparametric multifactor dimensionality reduction software (version 3.0.2).

**Results:**

Single locus analysis showed no association among the 23 SNVs with psychosis; however, gene-environment analysis was significant for the polymorphic loci rs12720071 and rs7766029 in *CNR1.* The best association models were the two-factor representing by the combination of *CNR1* rs12720071 with lifetime cannabis use (p<0.001), and *CNR1* rs12720071 with childhood trauma (p<0.05), both suggesting an increased risk of psychosis. Additionally, when considering the interaction of both environmental factors in the same model, we found *CNR1* rs7766029 to be associated with psychosis (p<0.001).

**Conclusions:**

Our study supports the hypothesis of gene-environment interactions for psychosis involving the T allele carriers of *CNR1* SNVs (rs12720071 and rs7766029), childhood trauma and lifetime cannabis use in psychosis.

**Disclosure of Interest:**

None Declared

